# Nanomedicine for the Diagnosis and Therapy of COVID-19

**DOI:** 10.3389/fbioe.2021.758121

**Published:** 2021-11-04

**Authors:** Yingruo Wang, Yuanping Hao, Shunxin Fa, Weiping Zheng, Changqing Yuan, Wanchun Wang

**Affiliations:** ^1^ Shandong University of Science and Technology, Qingdao, China; ^2^ Qingdao Stomatological Hospital Affiliated to Qingdao University, Qingdao, China; ^3^ School of Stomatology, Qingdao University, Qingdao, China; ^4^ York School, Monterey, CA, United States; ^5^ Department of Stomatology, The Affiliated Hospital of Qingdao University, Qingdao, China

**Keywords:** nanomaterials, COVID-19, SARS-CoV-2, theranostic nanomedicine, advanced materials

## Abstract

The coronavirus disease-2019 (COVID-19) pandemics caused by the severe acute respiratory syndrome coronavirus 2 (SARS-CoV-2) has been spreading around the world due to its high infection rate, long incubation period, as well as lack of effective diagnosis and therapy or vaccines, which is tearing global health systems apart. It is an urgent demand for point-of-care diagnosis and effective treatment to prevent the spread of COVID-19. Currently, based on the rapid development of functional materials with unique physicochemical features through advanced fabrication and chemical modification, nanomaterials provide an emerging tool to detect SARS-CoV-2, inhibit the interplay in the virus and host cell interface, and enhance host immune response. In our manuscript, we summarized recent advances of nanomaterials for the diagnosis and therapy of COVID-19. The limitation, current challenges, and perspectives for the nano-diagnosis and nano-therapy of COVID-19 are proposed. The review is expected to enable researchers to understand the effect of nanomaterials for the diagnosis and therapy of COVID-19 and may catalyze breakthroughs in this area.

## Introduction

**FIGURE 1:**
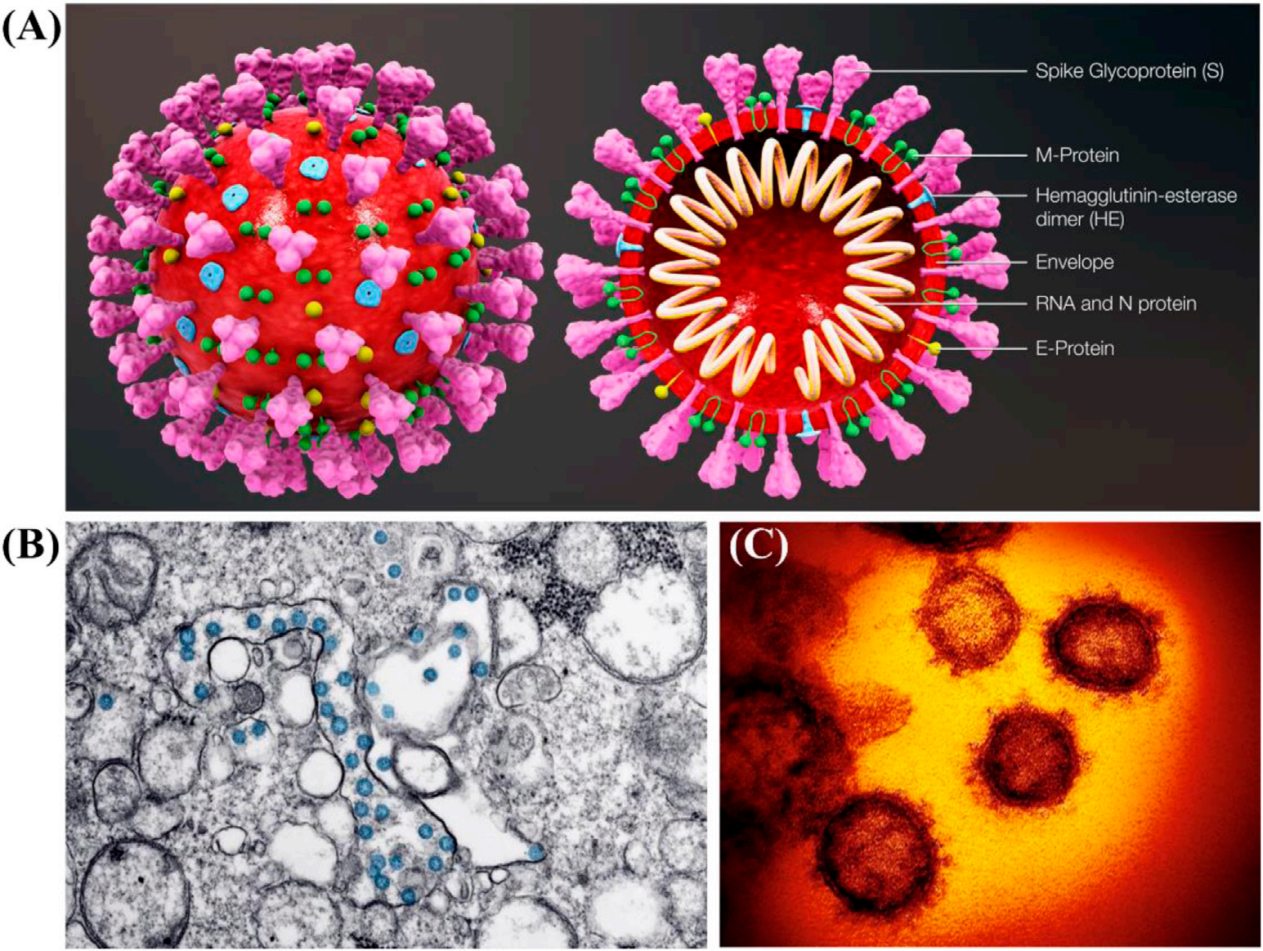
**(A)** Schematic diagram of SARS-CoV-2 and its cross-sectional representation with proteins (Scientific Animations, 2020). **(B)** Transmission electron microscope image of SARS-CoV-2 (CDC, 2020). **(C)** False colored images of SARS-CoV-2 (Kostarelos, 2020a).

**FIGURE 2:**
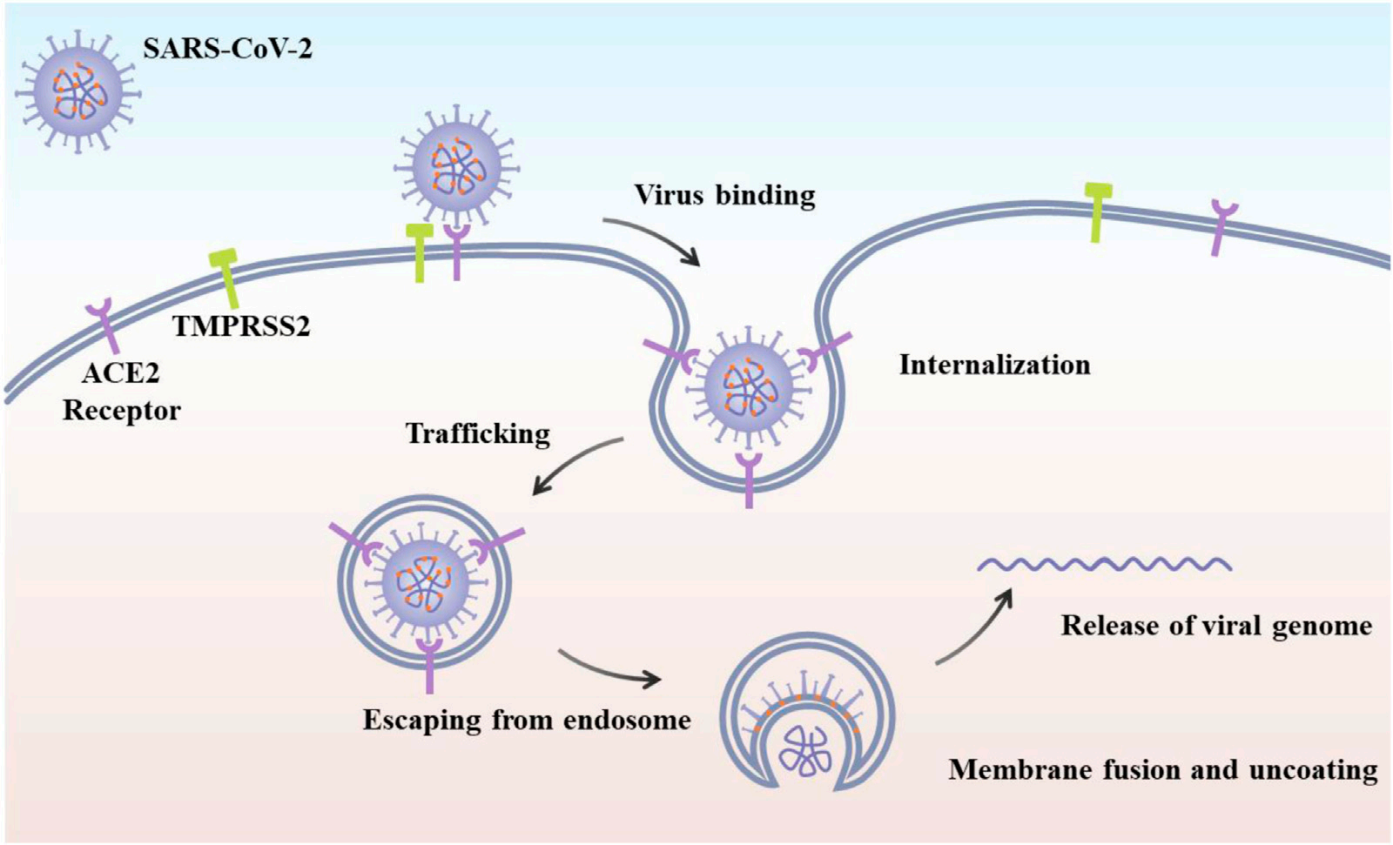
Schematic of SARS-CoV-2 viral life cycle. The initial attachment of SARS-CoV-2 to cells involves specific binding between the viral S glycoprotein and the cellular receptor, ACE2 (Yang et la., 2020).

The coronavirus infectious disease 2019 (COVID-19) was caused by the severe acute respiratory syndrome coronavirus 2 (SARS-CoV-2) with the magnificent nanostructure (Figure 1) (Shin et al., 2020; Weiss et al., 2020; Chang et al., 2020a; Kostarelos, 2020a). Since its first discovery in December 2019 in the Wuhan city of China, it has already infected millions of people worldwide and resulted in hundreds of thousands of deaths due to its high infection rate, long incubation period, as well as lack of effective and practical diagnosis and therapy or vaccines, which is tearing global health systems apart (Lai et al., 2020; Dima et al., 2020). The primary symptoms of SARS-CoV-2 infected patients include fever, dry cough, fatigue, and difficulty in breathing or maybe silent carriers (Cui et al., 2019; Chang et al., 2020b). In addition, SARS-CoV-2 has four major structural proteins, i.e., spike (S) protein, nucleocapsid (N) protein, envelope (E) protein, and membrane (M) protein (Figure 2) (Schoeman and Fielding, 2019; Phan, 2020; Wang et al., 2020). Particularly, the S protein plays a critical role in affecting cells, because it facilitates SARS-CoV-2 to determine the angiotensin-converting enzyme 2 (ACE2) and thereby invade into the host cell (Figure 2) (Gheblawi et al., 2020; Wang et al., 2020; Xia et al., 2020; Yan et al., 2020). Increasing evidence reveals that some patients with COVID-19 show severe organ damage, e.g., heart, liver, kidney, lung, the central nervous system, etc. (Batlle et al., 2020; Li et al., 2020; Zaim et al., 2020).

Its genome sequencing analysis indicates that SARS-CoV-2 has a single-stranded RNA genome (19 sequences), which is very similar to CoV, particularly β-CoVs (Guo et al., 2020; Neogi et al., 2020; Palestino et al., 2020). The availability of SARS-CoV-2 genome sequence is conducive to develop PCR kits to diagnose SARS-CoV-2 infected patients by the real-time reverse transcription-polymerase chain reaction (RT-PCR) (Ravi et al., 2020). Although the RT-PCR strategy is highly sensitive based in the detection of RNA, there are several defects as follows: 1) high cost and time-consuming (2–5 h), 2) sophisticated devices and complicated operation by highly skilled staff, 3) false-negative and false-positive results, and 4) a laboratory with biosafety level 2 or above (Nguyen et al., 2019; Palestino et al., 2020). Further, alternative techniques based on anti-bodies (serological testing) and Clustered Regularly Interspaced Short Palindromic Repeats (CRISPR) have been employed for the diagnosis of the SARS-CoV-2 infection. Chest computed tomography (CT) is also the optional imaging strategy for the detection of COVID-19 (Ye et al., 2020).

This inefficient strategy is exceptionally adverse for COVID-19 emergencies. Abbott designed a kit that achieved a 5-min rapid detection dependent on isothermal amplification of nucleic acid by testing the RNA-dependent RNA polymerase (RdRP) of COVID-19 (Carter et al., 2020). However, test kits developed (e.g., isothermal amplification tests, serological tests, etc.) also have some limitations, especially low accuracy (Lee et al., 2015). Developing rapid point-of-care and reliable diagnostic strategies is highly valuable in community clinics and emergency rooms and further prevents the spread of the SARS-CoV-2.

In addition, for the therapy of COVID-19, researchers are testing various drug formulations to treat SARS-CoV-2 infected patients (Cortegiani et al., 2020; Yang et al., 2020). Although the HIV drug and traditional Chinese medicine (TCM) had a positive effect on the treatment of COVID-19 (Luo et al., 2020; Ren et al., 2020), they still cannot cure COVID-19. Unfortunately, there are no vaccines or therapeutics available approved by the United States Food and Drug Administration (FDA) agency for treating patients with COVID-19. The research and development cycle of vaccines generally requires years before they can be used widely due to the regulatory steps required to ensure their safety and efficacy.

It has been demonstrated that nanomaterials offer an emerging platform for the point-of-care diagnosis carriers for therapeutics, and vaccine development owing to their low toxicity, unique size, tunable charge, chemical modification capabilities, and so on (Adhikari et al., 2020; Chauhan et al., 2020; Mainardes and Diedrich, 2020; Manivannan and Ponnuchamy, 2020; Palestino et al., 2020; Shin et al., 2020; Weiss et al., 2020). For the nano-diagnosis, nanomaterials can bind with target molecules to form a measurable signal, allowing the detection and identification of the virus (Bidram et al., 2021; Rashidzadeh et al., 2021; Vahedifard and Chakravarthy, 2021). The strategy is easy to operate and marketable, possesses stable, accurate, and highly sensitive features, as well as does not require specialized instrumentation. For the therapy, lipid nanoparticles containing mRNA vaccines have already reached Phase II clinical trials (Shin et al., 2020). In this review, we overviewed recent advances of nanomaterials for the diagnosis and therapy of COVID-19. The current challenges and perspectives for the nano-diagnosis and nano-therapy of COVID-19 are proposed. The review is expected to enable researchers to understand the effect of nanomaterials for the diagnosis and therapy of COVID-19 and may catalyze breakthroughs in this area.

## Nanomaterials for the Diagnosis of COVID-19

As mentioned, there are no vaccines or therapeutics available approved by the FDA for treating patients with COVID-19. Thus, rapid point-of-care nano-diagnosis of COVID-19 plays a key role in detecting COVID-19 patients to prevent further infection of the SARS-CoV-2 (Nguyen et al., 2019; Chan, 2020; Chauhan et al., 2020; Sharma et al., 2021). The tunable physicochemical properties of nanomaterials, such as size, shape, charge, and chemical functions (Cheng et al., 2013; Zhu et al., 2013; Liu et al., 2017), make them very useful in the diagnosis of COVID-19.

Previously, it was well-demonstrated that magnetic nanoparticles (MNPs) were valuable as nano-sensors (Mishra et al., 2018). Zhao *et al.* prepared carboxyl polymer-coated MNPs to combine the virus lysis and RNA binding steps into a single step (Zhao et al., 2020). This system can extract viral RNA from several samples within 20 min and 10-copy sensitivity. This method offers a simplified RNA extraction protocol to address the labor-intensive and time-consuming viral RNA extraction steps, and thus shows a promising alternative in the high-throughput molecular diagnosis of SARS-CoV-2. Taking into account the advantages mentioned above, another multifunctional nano-magnetic particle was designed and prepared to be used in RT-PCR for 2019-nCoV viral RNA extraction. Zinc ferrite NPs were prepared using the combustion synthesis method followed by modification with silica and carboxyl-functionalized polyvinyl alcohol (Somvanshi et al., 2020). The simple and cost-effective nature of this strategy may offer a capable substitute for traditional techniques to tackle the tedious time taking procedures and therefore demonstrates the ability of this method in detection of COVID-19.

Recently, the dual-functional plasmonic biosensor is used for the diagnosis of nucleic acid from COVID-19 (Qiu et al., 2020). This system includes the sensing transduction of localized surface plasmon resonance (ISPR) and the effect of the plasmonic photothermal (PPT). One plasmonic chip, which consists of Au-S bonding between thiol-cDNA receptor of RNA-dependent RNA polymerase (RdRp) and two-dimensional gold nanoislands (AuNIs), polyprotein ORF1ab, or the E gene sequence, can develop fast and delicate recognition of nucleic acids by enhancing the hybridization kinetics of matching strands.

To detect and monitor the progression of COVID-19, Chen and co-workers reported a rapid point-of-care lateral flow immunoassay based on lanthanide-doped polystyrene nanoparticles to identify anti-SARV-CoV-2 IgG in human serum in 10 min (Chen et al., 2020). Importantly, it was found that the outcome satisfies the demand for clinical diagnostic kits. Also, the strategy could be used to monitor the progression of COVID-19 and evaluating the response of SARS-CoV-2 infected patients to treatment.

Further, to avoid interference from other viruses, Moitra and co-workers developed gold nanomaterials combined with the antisense oligonucleotides specific for N-gene of SARS-CoV-2 viral genome to selectively detect positive COVID-19 patients within 10 min by a colorimetric assay (Moitra et al., 2020). Also, the anti-interference performance of the biosensor above was demonstrated that no significant change in absorbance was detected with the Middle East respiratory syndrome (MERS)-CoV RNA. This work describes an accurate, highly sensitive, and naked-eye detection of SARS-CoV-2 without the requirement of any special laboratory facilities.

## Nanomaterials for the Therapy of COVID-19

Nanomaterials provided a powerful platform to neutralize different viral infections, such as SARS or MERS coronaviruses (Abd Ellah et al., 2020; Barar, 2020; Bonam et al., 2020; Shapiro, 2021; Varahachalam et al., 2021). Thus, the use of nanomaterials displays great potential in developing novel therapeutic strategies for the therapy of COVID-19.

As mentioned in the introduction, the S protein of SARS-CoV-2 plays a critical role in its infection mechanism, which is similar to MERS-CoV. Based on the interaction between S protein and ACE2 on the host cell membrane, already existing antiviral nanomaterials can be used to treat COVID-19. For instance, the effective heptad repeat 1 (HR1) peptide inhibitor that is Pregnancy Induced Hypertension (PIH), can interrupt HR1/HR2-mediated membrane fusion between MERS-CoV and host cells. Huang and co-workers demonstrated that PIH released from Au nanomaterials possessed significant enhanced viral inhibitory ability than free PIH (Itani et al., 2020). Furthermore, this PIH/Au nanomaterial display high stability with potential applications for similar coronaviruses. In addition to kill the virus inside the body, nanomaterials can prevent the virus entry into the cells. Also, Łoczechin *et al.* developed boronic acid ligands conjugated with carbon quantum dots (CQDs) to inhibit the interaction between protein S-receptor and host cell membrane, inhibiting the virus entry into the host cells (Łoczechin et al., 2019). Recently, inhaled silver nanoparticles (Ag NPs) were used as a first-line approach for the treatment and prevention of COVID-19 infection progression (Sarkar, 2020; Zachar, 2020). The antiviral effect of Ag NPs may be attributed to the attachment of Ag NPs to RNA virus surface glycoproteins, stopping the virus from integrating into host cells.

Copper is known for its antimicrobial and antiviral activity since ancient times, and more lately, the Cu effectiveness to deactivate coronaviruses recommends possible alike effectiveness towards SARS-CoV-2. The deactivation mechanism is due to damage of viral proteins and lipids (Doremalen, 2020).

Also, increasing evidence demonstrated that nanomaterials served as potential tools for immune modulation to activate the immune response against a pathogen. For example, graphene oxide modified with amino groups mediated the signal path of STAT1/IRF1 interferon in T cells, inducing the expression of T cell chemoattractants (Orecchioni et al., 2017).

As reported, SARS-CoV-2 has a size of approximately 125 nm and be considered as natural nanomaterials (Kostarelos, 2020b). Nanomaterials that mimic the intrinsic immunostimulatory characteristics of viruses, enable the design of next-generation vaccine development. A messenger RNA (mRNA)−lipid nanoparticle vaccine has been tested to fight with SARS-CoV and MERS (Garber, 2018; Jackson et al., 2020). Particularly, McKay *et al.* developed a self-amplifying RNA encoding the SARS-CoV-2 S protein loaded in a lipid nanoparticle as a vaccine to stimulate high neutralizing antibody titers in mice. Their work offers new insight into the development of vaccines and the evaluation of immunogenicity to accelerate the translation of nanomaterial-based vaccines from the bench to the clinic (McKay et al., 2020).

## Limitation

Although nanomaterials have shown great potential in the diagnosis and therapy of COVID-19, studies have demonstrated that nanomaterials have the actual risk to cause detrimental actions to human health (i.e., nanotoxicology) (Singh et al., 2019; Miller and PolandNanotoxicology, 2020; Yu et al., 2020). One of the most frequently nanomaterial-related toxicities is oxidative stress responses caused by reactive oxygen species (ROS) generation, which can further induce pathophysiological effects, e.g., genotoxicity, inflammation, and fibrosis, etc. (Kim et al., 2015; Lujan and Sayes, 2017). The interactions between nanomaterial interface and cell, mediated by ROS, can damage the cell membrane, denature protein, as well as result in lipid peroxidation and alteration of calcium homeostasis, causing mitochondrial damage, immune cell activation, and nicotinamide adenine dinucleotide phosphate (NADPH) oxidase system (Fu et al., 2014; Lujan and Sayes, 2017; Yang et al., 2019; Yu et al., 2020). Most of nanomaterial intrinsic characterizations can catalyze ROS generation (Yang et al., 2019). Also, the physicochemical features of nanomaterials, e.g., morphology, size, charge, and component, affect the production of ROS and nanomaterial-induced damage (Yang et al., 2019; Yu et al., 2020). In addition to ROS, nanomaterials also result in reactive nitrogen species-mediated damage (Manke et al., 2013; Ferreira et al., 2018). Therefore, in terms of nanotherapeutics for COVID-19, a rigorous regulatory approval process must be performed to ensure safety and efficacy.

## Conclusion and Outlook

So far, no FDA-approved drugs are available for combating COVID-19, and particularly vaccines are under clinical trials. Increasing evidence indicates that nanomaterials could offer new insights to develop novel delivery systems or kill viruses directly. Also, nanomaterials can be used to detect virus-infected patients. In our manuscript, we summarized the recent progress of nanomaterials for the diagnosis and therapy of COVID-19. The inherent chemicophysical properties of nanomaterials, e.g., low toxicity, ultra-small dimension, high specific surface area, chemical modification capabilities, and high reactivity, play a critical role in point-of-care diagnosis and intriguing nanosystems for COVID-19 treatment. With the rapid development of engineered nanomaterials for advanced diagnosis and therapy, it is expected that nanomedicine would have a great impact on the in-depth of COVID-19 in the near future.

However, to accelerate the translation of nanomaterial-based outcomes from the bench to the clinic, more studies are needed to be performed. First, an extensive body of work is required to elicit the underlying mechanisms of the interactions between nanomaterial interface and SARS-CoV-2, tracing a reasonable design of COVID-19 therapeutics. Second, to improve the efficiency of COVID-19 therapeutics *in vitro* and *in vivo*, high-throughput strategies are expected to be designed. This strategy has many advantages, e.g., cost and time-efficiency, combining multi-parameter on a single system, and minimizing methodological or systematic errors. The big data from high-throughput strategies would provide a better understanding of the interplay between nanomaterial features and SARS-CoV-2. Finally, advanced technologies (e.g., artificial intelligence and other computation tools) could greatly shorten the lengthy process of high-performance nanomaterial discovery against SARS-CoV-2.
